# Microwave Ablation for Osteoid Osteoma in a Young Patient: A Case Report

**DOI:** 10.7759/cureus.61332

**Published:** 2024-05-29

**Authors:** Elijah Skarlus Doelakeh, Induni N Weerarathna, Anurag Luharia

**Affiliations:** 1 Anesthesia, Datta Meghe Institute of Higher Education and Research, Wardha, IND; 2 Biomedical Sciences, Datta Meghe Institute of Higher Education and Research, Wardha, IND; 3 Radiology, Datta Meghe Institute of Higher Education and Research, Wardha, IND

**Keywords:** computed tomography (ct), rural healthcare, minimally invasive, radiofrequency ablation, osteoid osteoma

## Abstract

Osteoid osteoma (OO) is a common benign ossifying lesion that is most prevalent among youth. Usually, it attacks the diaphyseal or metaphyseal bones that are tubular. The common hallmark of muscle pain is the reported occurrence of night pain that is nearly always present, yields satisfactory responses from nonsteroidal anti-inflammatory medications, and may be joined by complaints regarding physical activities. Also, it shows typical signs of study procedures like computed tomography (CT) and magnetic resonance imaging (MRI). A nidus, which is the primary marker in the diagnostic formation of shadowed images, is a crucial sign of an OO. This source is usually portrayed as an oval lytic lesion, measuring 1 cm flat and surrounded by a region of reactive ossification. It is laborious to diagnose OO since the condition is frequently confused with many other ones, and testing and therapy may be delayed and complicated as a result. There are still few studies on OO diagnosis and distinguishing of surrogate conditions. Unfortunately, either ablation or resection can be said to be the cure. Improved detection of OO shows the possibility for prompt diagnosis, fewer patient discomfort and side effects, less cost involved in unnecessary treatments, and a rightly diagnosed condition.

## Introduction

Osteoid osteoma (OO) is a benign bone-forming tumor. A small radiolucent nidus less than 1.50 cm in diameter produces high levels of prostaglandins. It typically occurs in patients during the second decade and affects the lower extremities. The most common site is the proximal femur. Patients with OO typically feel escalating pain, which worsens at night [1]. Plain X-rays are utilized to determine the condition. Patients with OO frequently report edema, restricted range of motion, and, most notably, nocturnal pain. The extreme pain associated with OO has been linked to increased prostaglandin production, which can reach levels 30 times higher than in normal bone. The pain-increased prostaglandin production from within the nidus causes severe inflammation and discomfort [2]. This case report documents the diagnosis and management of OO in a 17-year-old male presenting with progressive right thigh pain. We aim to highlight the challenges faced in a rural setting, emphasizing the utilization of radiofrequency ablation as a minimally invasive treatment option [3].

The patient's case was investigated through medical records (examined from October 27, 2023, to January 3, 2024) and imaging archives (collected from the surgery at Acharya Vinoba Bhave Rural Hospital (Sawangi Meghe)) that underwent microwave ablation for OO. The etiology was based on both clinical and radiological examinations; thereby, the patient was referred to a specialized surgical center to relieve the pain as the conventional approaches with drugs had no outcome. It is interesting to note that there is a small cortical lesion of size 3 mm by 2 mm within the lateral aspects of mid shaft of the femur with surrounding bone marrow edema, cortical thickening, and cortico-periosteal edema. Marrow edema noted in adjacent diaphysis suggested hyperemia. Technical skill is required to accompany the antenna into the nidus of the lesion at its periphery and hit the intended ablation heat. Pre- and post-ablation clinical evaluations were used at the one-month follow-up to measure the percentage of pain reduction, with clinical success being the complete pain alleviation with no medication needed. Every recording of problem-related data is made to document its occurrence and evaluate the level of its manifestations.

## Case presentation

A 17-year-old male, with no past medical history, reports right thigh pain of three months duration, which has built up gradually. However, he talks about the pain becoming more severe and then migrating to his right leg, mainly to his lower part. He rates his pain as moderate, and it increases mainly when he walks and does some physical work. Pain is improved by resting. The patient reported trauma to the right thigh five months ago. X-ray of right thigh with knee AP and lateral view shows s/o periosteal reaction with cortical thickening and sclerosis. Magnetic resonance imaging (MRI) of the right thigh suggested a neoplastic lesion, likely OO. 

**Figure 1:**
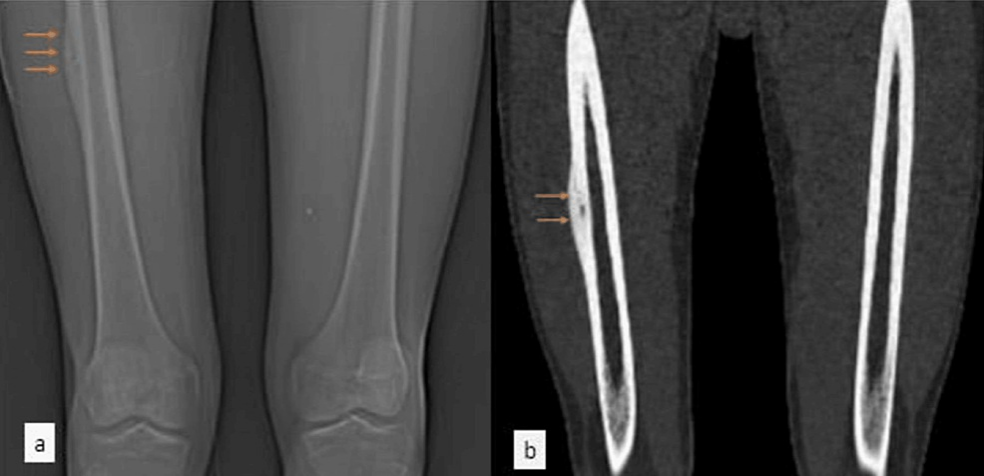
CT of the bilateral femur coronal sections of the topogram (a) and bone window (b) showing cortical-based lesion with a central osteolytic nidus suggesting OO CT, computed tomography; OO, osteoid osteoma

The patient's history revealed three months of insidious right thigh pain, aggravated by activity, and improved with rest. Imaging identified a lesion necessitating intervention. Following referral to the Interventional Radiology (IR) department, radiofrequency ablation was performed. The procedure encountered challenges in lesion localization, prolonging its duration. Successful ablation led to the patient's discharge. Figure 1 shows the computed tomography (CT) of the bilateral femur coronal sections of the topogram and bone window cortical-based lesion with a central osteolytic nidus suggesting OO.

## Discussion

OO are benign bone-forming tumors that are primarily found in adolescents and young adults and present with localized pain. Clinicians should have a strong clinical suspicion of OO in any young patient with back or neck pain, painful scoliosis, or radicular or referred-type pain in the lower limb or shoulder [4]. Its presentation characteristics can mimic that of a herniated disc or the lesion may cause radicular-like symptoms in the shoulders and arms. Furthermore, OO has been linked to an unidentified, rigid, or painful scoliosis, the latter in particular if the pain is referred to the concavity. Pain is a common symptom in the location of the tumor. Swelling can also develop in OO and is occasionally the sole manifestation. It is most common in patients with diaphyseal lesions [5]. Monarthritis, macrodactyly, clubbing, and pain-free proliferative swelling without reactive bone or bony lysis are all symptoms of hand engagement. OO of the hand and wrist are unusual. Although most involve the phalanges, they frequently manifest necessary clinical and radiologic indications [6].

They feature a comparable appearance to tumors in the foot and ankle. About 10% of intra-articular OO patients are affected by this sort of tumor. The hip, elbow, and ankle are the usual sources of the tumor [7]. However, OO is capable of taking appearance or being covered up by various other conditions, and this may result in a prolonged diagnostic and therapeutic process, along with associated complications [8]. This case highlights a 17-year-old male with a three-month history of progressively worsening right thigh pain, ultimately diagnosed as OO. The clinical manifestation of increasing pain, especially exacerbated at night, aligns with typical OO symptoms. The patient's presentation was consistent with the tumor's preferred location in the lower extremities, commonly observed in the proximal femur, as evidenced by our case [9]. Differential diagnosis considerations, such as stress fracture, subacute/chronic osteomyelitis, and osteoblastoma, underscore the importance of a thorough evaluation to distinguish OO from other potential causes of bone pain. Not considering the sickle cell trait and only focusing on the racial background of the patient reflects the complex classification of the mixed groups [10]. In this case, the management of OO varied depending on the availability of healthcare facilities, which influenced our patient's decision that radiofrequency ablation was chosen for his condition due to the difficulty of accessing healthcare services in a rural area [11].

This alternative promotes adopting minimally invasive surgery over traditional surgery due to its lower complication rate and better outcomes [12,13]. Notwithstanding the procedural hurdles that were encountered in this case, the successful completion of radiofrequency ablation in the IR department has equally brought to light that IR departments play a pivotal role in patient care through the provision of the best possible treatments. Moreover, the financial assistance of the social services team signifies the joint efforts required for the provision of optimal patient care, especially with a limited budget, which is characteristic of most health systems [14].

## Conclusions

The bone tumor, OO, with benign characteristics surely has a diagnostic and medical management problem. Here, RF ablation served as an effective solution in a farming region, overcoming patients' possible troubles with access to the healthcare system. Adopting minimally invasive surgery over traditional surgery can lower complication rates and treatment costs. After all, the case enlightens the clinical practice concerning OO management. It helps us understand the importance of individualized treatment and employing minimally invasive techniques, which in turn help increase the success rate of patient outcomes. We should keep practicing and explore other options constantly, which will help perfect the musculoskeletal tumor treatment techniques in the changing medical situation.
